# Low NAD^+^ Levels Are Associated With a Decline of Spermatogenesis in Transgenic ANDY and Aging Mice

**DOI:** 10.3389/fendo.2022.896356

**Published:** 2022-05-06

**Authors:** Mirella L. Meyer-Ficca, Alexie E. Zwerdling, Corey A. Swanson, Abby G. Tucker, Sierra A. Lopez, Miles K. Wandersee, Gina M. Warner, Katie L. Thompson, Claudia C.S. Chini, Haolin Chen, Eduardo N. Chini, Ralph G. Meyer

**Affiliations:** ^1^ School of Veterinary Medicine, Utah State University, Logan, UT, United States; ^2^ Department of Animal, Dairy, and Veterinary Sciences, College of Agriculture and Applied Sciences, Utah State University, Logan, UT, United States; ^3^ Signal Transduction and Molecular Nutrition Laboratory, Kogod Aging Center, Department of Anesthesiology and Perioperative Medicine, Mayo Clinic College of Medicine, Rochester, MN, United States; ^4^ Department of Anesthesiology and Perioperative Medicine Mayo Clinic, Jacksonville, FL, United States; ^5^ Department of Biochemistry and Molecular Biology, Johns Hopkins Bloomberg School of Public Health, Baltimore, MD, United States

**Keywords:** vitamin B3, niacin, nicotinamide, testis, aging, retinoic acid, spermatogonia, male fertility

## Abstract

Advanced paternal age has increasingly been recognized as a risk factor for male fertility and progeny health. While underlying causes are not well understood, aging is associated with a continuous decline of blood and tissue NAD^+^ levels, as well as a decline of testicular functions. The important basic question to what extent ageing-related NAD^+^ decline is functionally linked to decreased male fertility has been difficult to address due to the pleiotropic effects of aging, and the lack of a suitable animal model in which NAD^+^ levels can be lowered experimentally in chronologically young adult males. We therefore developed a transgenic mouse model of acquired niacin dependency (ANDY), in which NAD^+^ levels can be experimentally lowered using a niacin-deficient, chemically defined diet. Using ANDY mice, this report demonstrates for the first time that decreasing body-wide NAD^+^ levels in young adult mice, including in the testes, to levels that match or exceed the natural NAD^+^ decline observed in old mice, results in the disruption of spermatogenesis with small testis sizes and reduced sperm counts. ANDY mice are dependent on dietary vitamin B3 (niacin) for NAD^+^ synthesis, similar to humans. NAD^+^-deficiency the animals develop on a niacin-free diet is reversed by niacin supplementation. Providing niacin to NAD^+^-depleted ANDY mice fully rescued spermatogenesis and restored normal testis weight in the animals. The results suggest that NAD^+^ is important for proper spermatogenesis and that its declining levels during aging are functionally linked to declining spermatogenesis and male fertility. Functions of NAD^+^ in retinoic acid synthesis, which is an essential testicular signaling pathway regulating spermatogonial proliferation and differentiation, may offer a plausible mechanism for the hypospermatogenesis observed in NAD^+^-deficient mice.

## Introduction

Associated with socioeconomic considerations, for example increased time needed for education and professional development, mean paternal age has increased over the past 44 years from 27.4 to 30.9 years (1). This is concerning because paternal age has been shown to negatively affect fertility, pregnancy rates and children’s health (2, 3). How exactly the aging process exerts its negative effects on male fertility is not clear, because of its pleiotropic effects on the body, including the testis (4, 5). Although underlying mechanisms are not yet well understood, one of the hallmarks of aging is a steady decline of cellular, tissue and plasma NAD^+^ concentrations, observed during chronological aging in humans, worms, flies, and mice (6–10). NAD^+^ and NADP^+^, and their reduced forms NADH and NADPH, are important coenzymes for most cellular redox reactions, and as such essential for maintaining cellular metabolism and respiration. In addition to its function as a redox cofactor, NAD^+^ is also consumed by enzymes involved in chromatin modification, gene regulation, and DNA repair, including poly(ADP-ribose) polymerases (PARP family of enzymes), as well as NAD-dependent protein deacetylases (sirtuins) and CD38 (11–13).

Unfortunately, the links between aging, low NAD^+^ levels and declining fertility are not well understood because systematic investigations have been hampered by basic metabolic differences present between laboratory rodents and humans in their ability to generate NAD^+^ from their diet.

In certain mammals, including humans, nicotinic acid (NA), nicotinamide (Nam) and Nam riboside (NamR), collectively referred to as niacin or vitamin B3, are the main nutritional precursors of NAD^+^ and its phosphorylated form, NADP^+^. Humans depend on dietary niacin as their main source of NAD^+^ and NADP^+^ precursors and can become niacin-deficient when their food lacks sufficient amounts of vitamin B3. Niacin deficiency is characterized by very low levels of NAD^+^ and in its most extreme form, pellagra, can be debilitating and even deadly, which is now rare in western countries. However, milder forms of clinical niacin deficiency are commonly seen with increasing age, and in cancer patients, alcoholics and people without access to quality food (14, 15). While this may be clinically relevant on its own, it is unlikely that a lack of dietary vitamin B3 intake is at the root of age-related NAD^+^ decline. Instead, age-related increases in the activity of NAD^+^-consuming enzymes such as PARP1 and CD38, or potential mitochondrial dysfunction, or both, provide a more plausible explanation [(12, 16, 17), reviewed in (18)].

**Figure 1:**
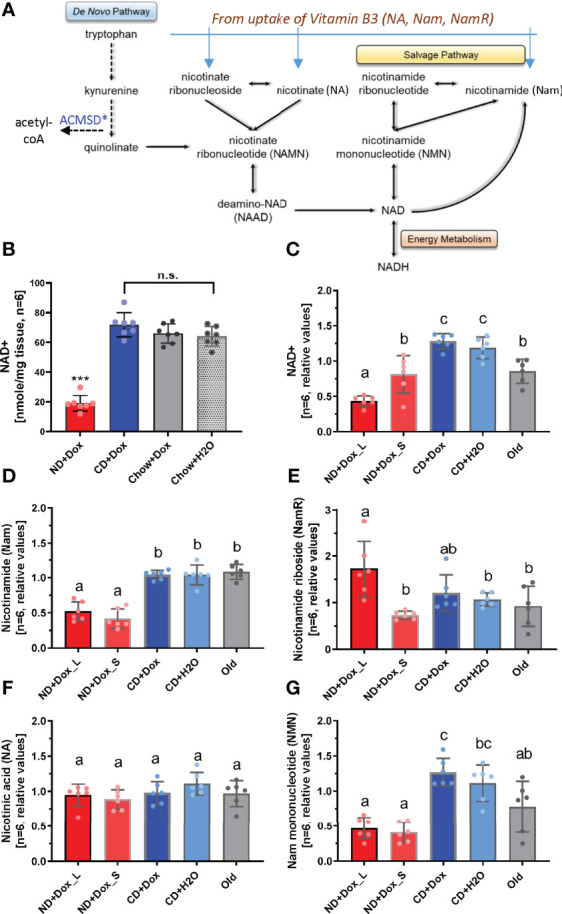
Dietary niacin deficiency altered NAD metabolite profiles in ANDY mice. ANDY mice were kept on niacin-deficient (ND) for up to 12 weeks (ND+Dox_S) or longer (ND+Dox_L) or on control diet with Dox for ACMSD transgene induction (CD+Dox) or without Dox (CD+H2O). Old mice were 31 months old. **(A)** The NAD *de novo* synthesis pathway from tryptophan can provide all of the NAD in wild-type rodents in absence of all other dietary NAD precursors such as nicotinic acid (NA, nicotinate), nicotinamide (Nam), or nicotinamide ribonucleotide (nicotinamide riboside, NamR). Dox-mediated induction of a human ACMSD transgene overexpression diverts the central kynurenine pathway in the liver and kidney from NAD^+^ production towards acetyl-CoA formation, and ultimately makes these mice dependent on dietary niacin to maintain tissue NAD^+^ levels similar to humans **(B)** Testicular NAD^+^ levels decline in ANDY mice on vitamin B3 (niacin) – free diet (ND). Data were generated using enzymatic cycling assays. **(C)** Metabolomic analyses confirms data in B, and indicates that a short-term period on ND diet (ND+Dox_S) results in a milder decline of NAD^+^ that is comparable to old mice. **(D)** Nam levels declined already after short-term dietary niacin-deficiency, while NamR **(E)** values appeared to increase again in mice with long-term niacin deficiency. Hypothetically, the latter may result from loss of spermatogonia, spermatocytes and spermatids that occurs at later stages of NAD^+^ decline. **(F)** Whole NA (nicotinate/nicotinic acid) did not change on the ND diet, while NMN **(G)** was significantly lowered in both short-and long-term ND fed mice compared to controls, but not old mice. Identical letters indicate group categories that are not significantly different from each other; different letters indicate statistically significant differences (One-way ANOVA with Tukey’s multiple comparison analysis, p<0.05 considered a significant difference; ***p > 0.001).

Physiological effects of low NAD^+^ status and their potential impact on male fertility have been difficult to study because of a lack of suitable animal models. Wild-type laboratory rodents are able to completely satisfy their NAD^+^ needs by metabolizing tryptophan (Trp) to NAD^+^ *via* the kynurenine (*de novo* synthesis) pathway and, unlike humans, do not depend on intake of dietary niacin. In order to address this problem and to investigate the impact of low NAD^+^ levels as a potential factor contributing to the decline of fertility in aging males, we therefore generated mice with tetracycline-inducible overexpression of a transgene encoding the enzyme human aminocarboxymuconate semialdehyde decarboxylase (hACMSD) to create a mouse model of human-like NAD^+^ metabolism (ANDY, acquired niacin dependency) (19). In this mouse, hACMSD overexpression diverts the central kynurenine pathway in the liver and kidney to produce acetyl-CoA instead of NAD^+^ which makes the animals dependent on dietary niacin intake as the main source of NAD+ synthesis, similar to humans (19) (**Figure 1A**). ANDY mice with hACMSD overexpression reproducibly become NAD^+^-deficient in various tissues over the course of 6 weeks on a defined diet that is devoid of niacin (ND diet), but not on a control diet that is chemically identical to ND but supplemented with 30 mg/kg nicotinic acid (CD diet). Previous data showed that ANDY mice had significantly lower NAD^+^ and NADP^+^ levels in blood, liver, and other tissues when they received a niacin-free ND diet and doxycycline (Dox, a water-soluble tetracycline) in their drinking water (19). If maintained at very low NAD^+^ levels, male ANDY mice sired smaller litters than control males (data not shown).

The goal of the current study has therefore been to investigate the impact of NAD^+^ deficiency on spermatogenesis in young adult ANDY mice to test the hypothesis that low NAD^+^ levels have a negative impact on male fertility, independent of chronological age.

## Materials and Methods

### Animal Model and Induction of NAD Deficiency

Details of the generation of the transgenic animal model C57BL/6J-Gt(Rosa)26Sor^tm1(rTTa*M2)Jae^Col1a1^tm6(tetO-hACMSD)MMF^ and the biochemical basis of NAD-dependency in these mice has been described previously (19). Briefly, administration of doxycycline, a water-soluble tetracycline, in the drinking water induces overexpression of the human aminocarboxymuconate semialdehyde decarboxylase (hACMSD) gene. Increased ACMSD activity renders these transgenic mice dependent on dietary niacin uptake in a manner similar to humans. In the absence of dietary niacin, these ANDY mice become measurably NAD^+^ deficient in blood and body tissues (19). Mice were bred and housed under standard conditions. Transgene expression was only induced in adult mice during the feeding trials. Breeding, postnatal and pubertal development occurred in the absence of doxycycline-induced transgene overexpression and on normal, niacin-containing chow diet. Animal studies and experimental procedures were approved by the Institutional Animal Care and Use Committees (IACUC protocol number 10056) of Utah State University and of Mayo Clinic, Rochester, Minnesota.

### Defined Feeds and Feeding Trials

Standard chow diet was Teklad Rodent diet 8604 (24% crude protein, 63 mg/kg niacin, Envigo, Madison, WI, USA). Niacin-deficient diet (ND, TD.140376) and control diet (CD, TD.140375) were defined, purified diets compounded by Teklad laboratory animal diets (Teklad Custom Diets, Envigo) as modifications of AIN-93G standard chow (19). Both, ND and CD contained 10% alcohol-washed casein as a vitamin-free protein source, either without niacin (ND) or with 30 mg/kg niacin (CD).

Age- and weight-matched animals were randomly assigned for the experiments when they were sexually mature, young adult mice between 7-14 weeks of age. During each feeding trial, mice were either fed ND or CD. Doxycycline (Sigma Aldrich, D9891; Alfa Aesar J6057922) was added to the drinking water [2 mg/ml] to induce ACMSD expression. Drinking water was changed twice per week. Durations of feeding trials are indicated in the results and figure legends. In recovery studies, animals were first kept on ND+Dox for the indicated time interval to induce NAD+-deficiency, then switched back to CD for the indicated recovery time.

At termination of each study, animals were euthanized, heparinized blood samples and tissues were collected rapidly, tissues weighed, snap frozen in liquid nitrogen and stored at -80°C until further analyses. Samples for histology were fixed immediately. Sperm numbers were determined in epididymal sperm isolated from the cauda epididymides and the vas deferens using a Neubauer hemocytometer.

### Histology and Evaluation

Tissues for histological analyses were fixed in Bouin’s solution (Sigma Aldrich, HT10132) or 10% neutral buffered formalin (Sigma Aldrich, HT501128-4L) immediately after tissue collection. Paraffin embedding, sectioning and hematoxylin eosin staining was performed by the Utah Veterinary Diagnostic Laboratory’s histology core facility according to standard histological procedures.

Testicular tubules were analyzed for abnormalities in a blinded manner by an individual that had been trained in identifying spermatogenic stages in the mouse. Hematoxylin/eosin stained paraffin sections of testes were analyzed using bright field microscopy (Axio Scope A.1, Zeiss, Jena, equipped with AxioVision software). Tubules were classified as abnormal if they were missing a complete layer of cells that are normally present in a given tubular stage and that are used for classification of tubules, e.g. the absence of spermatocytes or round spermatids in stages I-VIII, or absence of spermatocytes and/or condensing spermatids in stages IX-XII, and/or if absence of several cell layers prevented stage identification. One hundred tubules were evaluated per animal and testis section, and statistical analysis was performed using 1-way ANOVA with Tukey’s multiple comparison test.

### NAD Measurements

Testicular tissue NAD^+^ was quantified using an enzymatic cycling assay method described previously (19–21). Briefly, frozen testis tissue was lysed in NaOH, and neutralized with H_3_PO_4_. Protein was removed by HClO_4_ precipitation, and supernatant was treated with KOH. NAD^+^ was quantified in the supernatant in a 96-well microplate format on a SpectraMax Plus 384 plate reader (Molecular Devices, Sunnyvale, CA). All chemicals were from Sigma, Aldrich (St Louis, MO).

### Testosterone Measurements

Testicular testosterone was quantified by radio immuno-assay as described previously (22, 23). In brief, snap-frozen pieces of testicular tissue were extracted in 2 ml of assay buffer and testosterone was measured using standard RIA procedure with a testosterone specific antibody (ICN Biomedicals, Costa Mesa, CA) and ^3^H-T (NEN Life Science Products, Boston, MA).

### Quantification of Metabolites in Testicular Tissue

Testicular testosterone, nicotinamide adenine dinucleotide (NAD), nicotinamide (Nam), nicotinamide riboside (NamR), nicotinic acid (NA) and Nam mononucleotide (NMN) were quantified with Ultrahigh Performance Liquid Chromatography-Tandem Mass Spectroscopy (UPLC-MS/MS) on the Metabolon Platform (Metabolon, Norrisville, NC). Frozen testis samples were prepared using the automated MicroLab STAR^®^ system (Hamilton), proteins were precipitated with methanol followed by centrifugation. The resulting extract was analyzed by two separate reverse phase (RP)/UPLC-MS/MS methods with positive ion mode electrospray ionization (ESI), by RP/UPLC-MS/MS and by HILIC/UPLC-MS/MS, both with negative ion mode ESI. UPLC-MS/MS was performed on a Waters ACQUITY UPLC and a Thermo Scientific Q-Exactive high resolution/accurate mass spectrometer interfaced with a heated electrospray ionization (HESI-II) source and Orbitrap mass analyzer operated at 35,000 mass resolution. The sample extract was dried, then reconstituted in solvents compatible to each of the four spectroscopy methods. Each reconstitution solvent contained a series of standards at fixed concentrations to ensure injection and chromatographic consistency. The MS analysis alternated between MS and data-dependent MSn scans using dynamic exclusion. The scan range varied slighted between methods but covered 70-1000 m/z. Raw data was extracted, peak-identified and QC processed using Metabolon’s hardware and software. Compounds were identified by comparison to library entries of purified authenticated standards, and peaks were quantified using area-under-the-curve.

### Graphing and Statistical Analyses

GraphPad Prism software versions 7.04 & 9.2.0 (GraphPad Software, San Diego, CA) were used for graphing and statistical analyses (One-way ANOVA, Tukey’s multiple comparison, Welch’s t-test, Pearson Correlation analysis; p<0.05 was considered significant).

## Results

### ANDY Mice on Niacin Free Diet Have Significantly Reduced Testicular NAD^+^

In the absence of dietary niacin, blood NAD of ANDY mice declined steadily over time a time span of 6 weeks, and then remained at significantly lower levels compared to those in control animals (**Supplementary Figure 1**). Similar to blood, testes of ANDY mice became niacin-deficient, i.e. had significantly decreased NAD^+^ tissue concentrations. NAD^+^ levels in ANDY mice that were maintained on ND+Dox for 24 weeks dropped to about 1/3 of the NAD^+^ content measured in ANDY mice fed niacin-containing CD diet or chow (**Figure 1B**), as measured using a sensitive enzymatic cycling assay (**Supplementary Table 1**). Similar changes were observed using comparative metabolomics analyses of testes from animals fed ND for 24 weeks (long-term, ND+Dox_L) or 12 weeks (short-term, ND+Dox_S). The metabolomic LC-MS/MS quantification confirmed the significant lowering of testicular NAD^+^ levels in the ND+Dox_L group (long-term on ND diet, i.e. >12 weeks, one-way ANOVA, p-values from <0.0001 to 0.0052 with Tukey’s multiple comparison test, **Figure 1C**). Compared to this group, NAD^+^ levels were higher in ANDY mice kept on ND for 12 weeks (ND+Dox_S, p=0.0052), but still significantly lower than the control groups (p-values from 0.0004 to 0.0055). NAD^+^ contents in controls CD+Dox and CD+H2O were not significantly different from each other, indicating that ACMSD overexpression and doxycycline administration on their own did not have any measurable effect on NAD^+^ levels in the testis. Interestingly, NAD^+^ content in the testes of the ND+Dox_S group was not significantly different from that of old mice at 31 months of age. Nam levels were low in both short- and long-term ND groups (**Figure 1D**). Unexpectedly, NamR levels were significantly higher in the ND+Dox_L group than ND+Dox_S(p=0.0011), but not significantly different from the CD+Dox control and the old mice (**Figure 1E**). NA values did not vary between the different treatment groups (**Figure 1F**). Similar to NAD^+^ and NamR, NMN was not significantly different between mice in the ND+Dox_S group and old mice (**Figure 1G**).

Taken together, ACMSD overexpression in combination with niacin-free feed significantly lowered testicular NAD^+^ levels of ANDY mice, which is also reflected in an altered NAD^+^ metabolite profile. Moreover, the NAD^+^ levels created in ANDY mice of the ND+Dox_S group were similar to those in old mice at 31 months of age.

### Declining Testicular Weight and Sperm Counts in NAD^+^-Deficient ANDY mice

**Figure 2:**
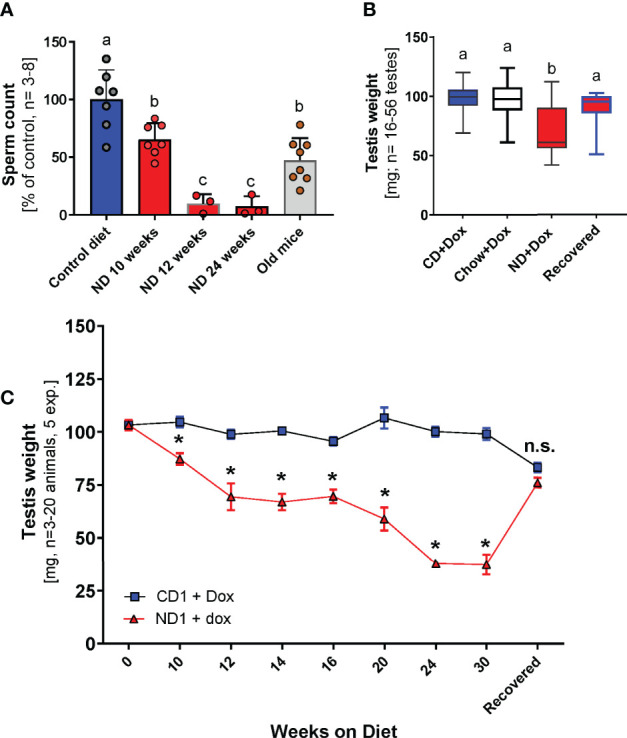
Testicular decline when NAD+ levels are low. **(A)** Sperm counts of old mice and ANDY mice on ND+Dox diet (10 weeks) were significantly lower than controls, but not statistically different from each other. Sperm counts of ND+Dox fed mice were dramatically lower after 12 weeks and remained low after that (one-way ANOVA with Dunett’s multiple comparison analysis comparing % of sperm count at indicated time points with sperm counts at start of study, only sperm from ND diets were significantly different; P-values range was 0.0001 to 0.01). **(B)** Testes of ANDY mice on ND diet were significantly smaller on average (1-Way ANOVA, p= 0.0001 Tukey’s multiple comparisons: ND group is significantly different from recovered ND (9 weeks recovery on CD diet): p<0.0001; Chow:: p<0.0001 CD1: p<0.0001. ND recovered and CD- or chow-fed group chow were not significantly different from each other. **(C)** Testis weights significantly declined in ANDY mice on niacin-deficient (ND) diet over the course of 24 weeks compared to ANDY mice on control diet (CD, 30 mg/kg niacin). After changing ANDY mice that had initially been on ND diet to CD diet for 9 weeks, testis weights recovered in these animals and were no longer significantly different from the CD-fed group. Multiple t tests of row stats, significant difference in weeks 10-30, p<0.0039 and smaller; * indicates significant difference to the control value, values at time points 0 and after recovery not significantly different. Groups marked with the identical letter (a, b or c) were not significantly different from each other; differing letters indicate a significant difference; n.s. indicates no significant difference.

Sperm counts of mice that were kept on ND diet for ten weeks decreased significantly compared to control animals, and were similar to sperm numbers in old mice (**Figure 2A)**. After two additional weeks on deficient diet, sperm numbers declined abruptly (**Figure 2A**). Along with falling sperm counts, testes of mice in the ND+Dox group became significantly smaller than testes of any other treatment group as soon as 10 weeks on this diet, and continued to shrink until week 24 (**Figure 2B**). When recovered on the CD diet for 9 weeks, testis weights returned to normal values (**Figures 2B, C**). These results demonstrate that declining NAD^+^ levels resulted in testicular shrinkage that was reversed by niacin supplementation which restored NAD^+^ levels.

### NAD^+^-Deficiency Causes a Reversible Cessation of Spermatogenesis

**Figure 3:**
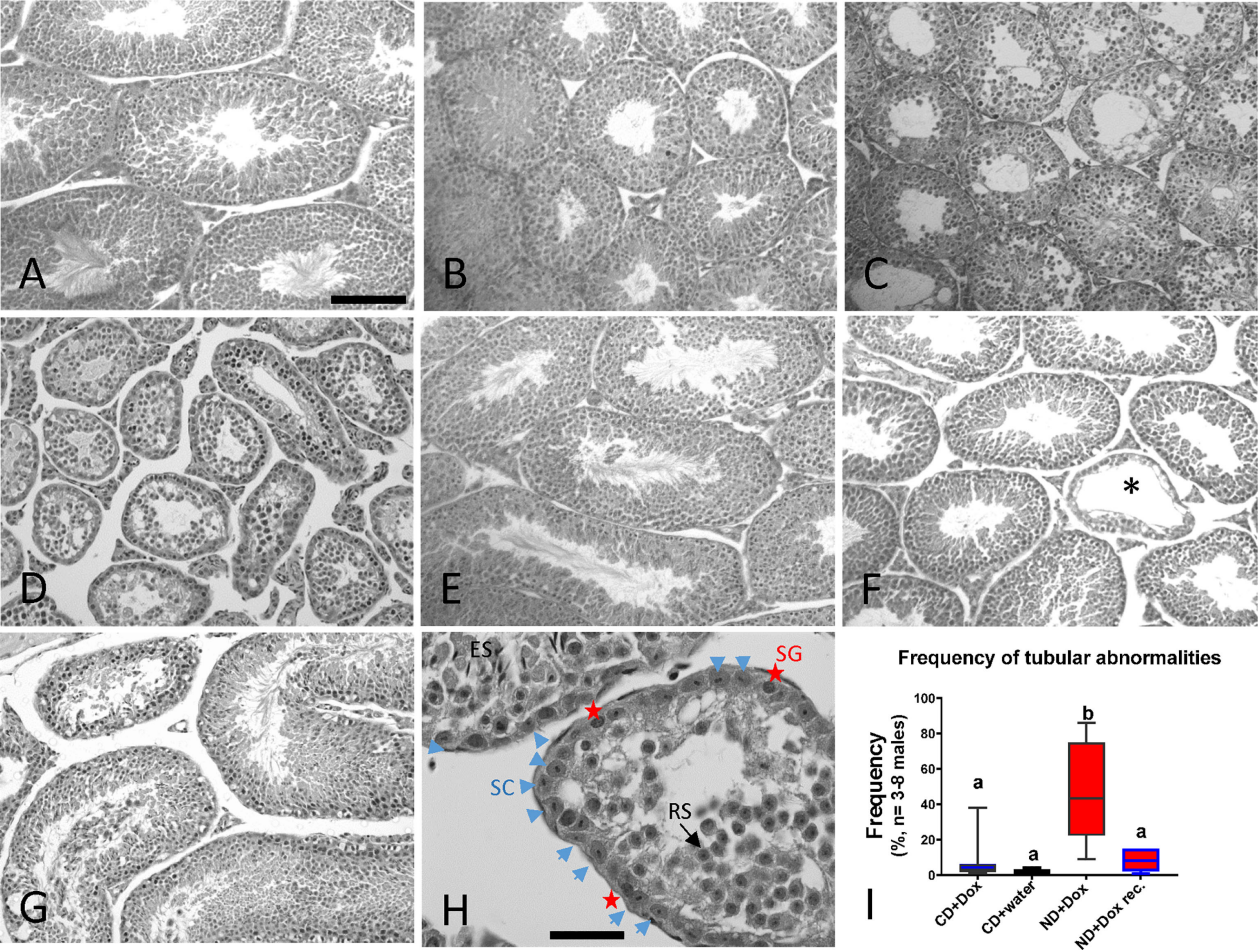
Impact of testicular NAD^+^ levels and male age on the seminiferous epithelium. Hematoxylin/eosin-stained testicular sections of testes from young adult male fed **(A)** CD+Dox control diet, **(B)** ND+Dox diet for 8 weeks, **(C)** ND+Dox for 14 weeks; damaged tubules **(D)** ND+Dox for 24 weeks; normal tubules +/- absent **(E)** ND for 24 weeks followed by 9 weeks of recovery on niacin-containing CD diet; tubules mostly restored. **(F)** Control testis at 20 months of age. Asterisk marks a tubule with abnormal seminiferous epithelium. **(G)** Control testis of 31 month-old mouse. **(H)** After 24 weeks on ND diet, seminiferous tubules are lined mostly by Sertoli cells (SC, blue arrow heads) interspersed with spermatogonia (SG, red stars), as identified by histological morphology of the cells. Tubular lumen contain mostly cells resembling round spermatids (RS, black arrow) and occasionally elongated spermatids (ES)). **(I)** NAD^+^-deficient testis contain significantly more tubules with abnormal composition of the seminiferous epithelium in testis of mice that were on indicated diets for 16 weeks (CD+Dox, CD+water, ND+Dox) or ND+Dox that were subsequently recovered on niacin containing diet for 9 weeks. One hundred tubules were evaluated per testis section, ANOVA with Tukey’s multiple comparison, b is significantly different from a, p=0.0003 to 0.001. Scale bar: 250 mm in a.-f., 40 mm in (g) & h.

Histological evaluation of the testicular shrinking process (**Figure 3**) in testis from animals on the niacin-free ND diet revealed progressive seminiferous epithelial defects compared to control animals on CD+Dox (**Figures 3A–D**). Seminiferous tubules showed a lack of ongoing spermatogenesis with severely decreased numbers of spermatogonia and spermatocytes, as well as an abnormal spatio-temporal organization (**Figures 3C, D**). Seminiferous epithelia of animals kept on ND+Dox for 24 weeks, followed by recovery on niacin-containing CD+Dox for 9 weeks, were restored to full cell complements, consistent with the observed reversal of testicular shrinkage (**Figures 2B, D**). Seminiferous tubules of older mice at 20 month-old appeared mostly normal, except for the appearance of sporadic abnormal seminiferous tubules (asterisk in **Figure 3F**), while seminiferous tubules in testes of 31 month-old mice displayed marked and frequent disorganization of the seminiferous tubules (**Figure 3G**). After 24 weeks on ND+Dox diet, seminiferous tubules were lined mostly by Sertoli cells and some spermatogonia, and contained cells that appeared to be mostly residual round and some elongated spermatids (**Figure 3H**). Quantification of abnormal tubules in testis sections after 16 weeks on the indicated diets showed that NAD^+^-deficient testes contained significantly more tubules with abnormal composition of the seminiferous epithelium than controls or mice that were first kept on ND+Dox diets for 24 weeks and then recovered on CD for 9 weeks (**Figure 3I**). Taken together, the histological results suggested that a lack of spermatogonial proliferation led to a paucity of promeiotic and meiotic germ cells, which together make up more than half of the testicular weight and size in a normal animal. The remarkable recovery of spermatogenesis and subsequent doubling of testicular volume to a normal state in animals recovered on CD diet further indicates that spermatogonial stem cells remained intact and capable of restoring full spermatogenesis once NAD^+^ levels returned to normal levels (**Figures 2B, C** and **3E**).

### NAD^+^-Deficiency Did Not Affect Testicular Testosterone Content, but Was Associated With Increased Testicular Retinol Concentrations

**Figure 4:**
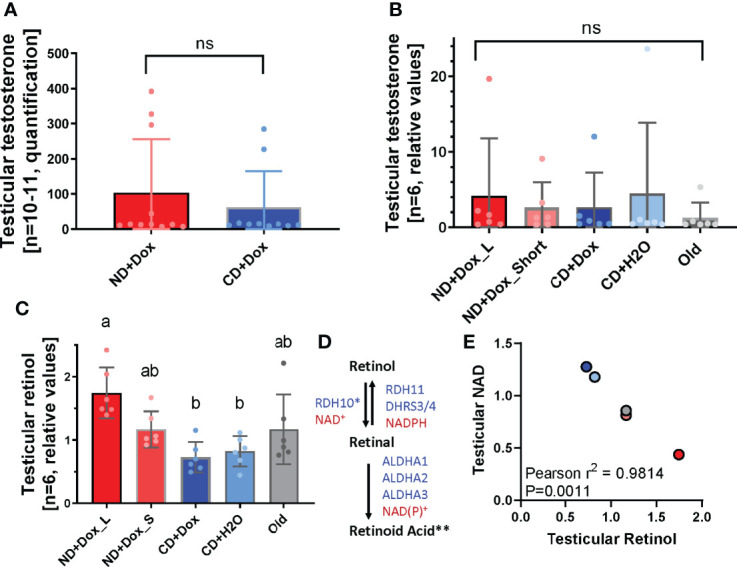
Unchanged testicular testosterone, elevated retinol levels in niacin-deficient ANDY mice with NAD+ decline. **(A)** Testicular testosterone levels were not significantly different between ND+Dox and CD+Dox groups (one-tailed unpaired students t-test, p=0.238). **(B)** Metabolomic analysis confirmed that result. **(C)** Mice in the ND+Dox groups had significantly elevated testicular retinol (vitamin A) concentrations (1-Way ANOVA, p= 0.0005; Tukey’s multiple comparisons: ND+Dox_L is significantly different from CD+Dox: p=0.0005; CD+H2O: p=0.0015, but not different from ND+Dox_S and old mice. Differences between CD+Dox, CD+H2O, ND+Dox_S and old mice did not reach statistical significance. Groups marked with the identical letter (a, b) were not significantly different from each other; differing letters indicate a significant difference; ns indicates no significant difference. **(D)** Retinol metabolism to form retinoic acid, which is an essential signal molecule in spermatogonial differentiation and proliferation and regulator of meiosis. Retinol (vitamin A) is transported into the testicular germ and Sertoli cells, and then converted into retinal by NAD^+^-dependent oxidation mediated by retinol dehydrogenase 10 (RDH10), which is the rate-limiting step (*) in the conversion to retinal. Alternatively, retinol can be converted to retinal in an NADPH-dependent fashion by RDH11, or by DHRS3 and DHRS4. Retinal is then further oxidized by aldehyde dehydrogenases ALDHA1/2/3 in an NAD(P)^+^-dependent way to form the active compound retinoic acid [**, after Gewiss, Topping and Griswold, 2019 (26)]. **(E)** Pearson’s correlation analysis reveals highly significant inverse correlation between testicular NAD (Y-axis) and testicular retinol (X-axis), with p=0.0011.

We initially hypothesized that the pronounced, but reversible, detrimental effect of low NAD^+^ levels on testicular function could result from impaired testosterone synthesis and metabolism. Unexpectedly, however, significant differences in testosterone levels due to individual NAD^+^ status could be detected neither by radioimmuno-assays (**Figure 4A**) nor by LC-MS/MS analyses (**Figure 4B**). Each diet group contained animals with markedly higher testosterone levels than those in their group mates, indicating that the ability to synthesize testosterone was not principally suppressed in any of the diet groups. Because retinoic acid (RA) signaling is essential for spermatogonial proliferation and differentiation, we used LC-MS/MS to analyze vitamin A metabolites in ANDY mice on different diets. Retinal and RA were not detectable using this method, but levels of the precursor molecule retinol were determined to be significantly elevated in ND+Dox_L, ND+Dox-_S, and old mice compared to control animals, with concentrations being the highest in the ND+Dox_L group (**Figure 4C**), suggesting a possible negative correlation of testicular retinol- and NAD^+^-levels (**Figure 1C**). Testicular retinol and testicular NAD content were highly significantly inversely correlated (**Figure 4E**). The accumulation of testicular retinol may be interpreted as resulting from a reduction or block of the rate-limiting step in the RA synthesis pathway that oxidizes retinol to retinal (**Figure 4D**), which is mediated by the NAD^+^-dependent enzyme retinol dehydrogenase (RDH10). Retinal is further oxidized to RA by aldehyde dehydrogenases ALDHA1/2/3, which are NAD(P)^+^ dependent enzymes as well. Therefore, NAD^+^ deficiency could lead to an inhibition of RA in the testis, potentially contributing to the observed spermatogonial proliferation and differentiation defect observed in ND+Dox mice.

## Discussion

The main results of this study are (i) that NAD^+^ deficiency can be produced in ANDY mouse testes on a niacin-free diet, and that the degree of this deficiency increases over time (**Figure 1**). To our knowledge, this is the first time this has been accomplished in a laboratory research animal. (ii) The degree of NAD^+^ decline that was achieved by keeping ANDY mice on niacin-free diet for 10-12 weeks was typical of an aging mouse (**Figure 1**). (iii) Low testicular NAD^+^ levels resulted in the attenuation of spermatogenesis and testicular atrophy due to impaired spermatogonial proliferation and differentiation (**Figure 3**). (iv) Recovery of mice on a niacin-containing control diet fully reversed testicular shrinkage and fully restored spermatogenesis (**Figure 3E**). Because NAD^+^ decline resulted in attenuation of spermatogenesis in ANDY mice, it may represent a link between low NAD metabolism as a hallmark of aging, and the decline of male fertility as males age.

Based on our data, low testosterone levels were not the determining factor for the observed hypo- and aspermatogenesis (**Figure 4**). However, the loss of mature germ cell stages in severely NAD^+^ depleted testes and overall seminiferous tubule histology was reminiscent of vitamin A-deficient males, where tubules appear to have only Sertoli cells and early stages of spermatogonia left in the tubular lumen (24). Vitamin A1 (retinol) is essential for spermatogenesis because it is the dietary precursor for RA synthesis (25) (**Figure 4i**). RA signaling is indispensable for spermatogonial proliferation and differentiation. If blocked by the inhibitor WIN 18,446 in adult rodents or humans, spermatogonial differentiation is disrupted and a vitamin A deficiency phenotype is created in the testis (24, 26–28). The rate-limiting step of RA synthesis is the oxidation of retinol to retinal by retinol dehydrogenase (RDH10), which is entirely dependent on the availability of NAD^+^ as a cofactor. In addition, the next step in RA synthesis is the conversion of retinal to retinoic acid, which is dependent on NADP^+^, whose levels are linked to cellular NAD^+^ stores (**Figure 4D**). This step, which is mediated by the aldehyde dehydrogenase (ALDHA) family of enzymes is also essential for testicular RA synthesis, and thus for the execution of spermatogenesis (27). Our finding that retinol appeared to accumulate to significantly higher levels in both NAD^+^-deficient and aging mice in a manner significantly inversely correlated with NAD^+^ levels (**Figure 4E**) therefore provides an intriguing clue that low NAD^+^ levels may block RA synthesis and thus cause the observed spermatogenic failure. However, additional investigations will be necessary to provide further confirmation of this hypothesis. Mechanisms underlying the aging process are still poorly understood, in part because effects of chronological aging are numerous and difficult to separate from environmental and intrinsic factors affecting a given individual over time. The NAD^+^ decline observed in aging animals and humans appears to be a consequence of the aging process, for example by means of failing mitochondrial activity, or through elevated consumption of NAD^+^ by PARP enzymes or elevated tissue activity of the NAD glycohydrolase CD38 (7, 16–18, 29–32). However, to what extent NAD^+^ decline itself may also be a driver of the aging process has remained an open question. The current study takes full advantage of the novel ANDY mouse model that allows for the first time that NAD+ levels in rodents can be lowered significantly, independent of the chronological age of the animal. The results of this initial investigation suggest that low and very low levels of NAD+ result in testicular decline in mice, similar to that observed in aging males. This finding suggests that NAD^+^ decline itself may promote aspects of the pathophysiology of aging.

NAD and NADP serve not only as an essential cofactor for enzymatic reaction in energy metabolism; they are also essential cofactors for several cellular mechanisms that protect the genome against DNA damaging insults, e.g. from reactive oxygen species (ROS). There is an age-related increases in ROS, the so-called “free radical theory of aging”, that is also evident in context of spermatogenesis and sperm quality (33–35). In fact, aging has been associated with reduce genetic quality in spermatogenic cells and sperm (36–38). Because NAD and NADP are required for both, maintaining a sufficient pool of the active antioxidant glutathione GSH, and for the enzymatic activity of PARP1, an important DNA repair factor, lower testicular NAD could potentially contribute to the aging-related accumulation of ROS and decline in sperm quality. S Investigations are currently underway to address this important question.

A potential limitation of the present study is that the degree of testicular NAD^+^-decline produced in ANDY mice that were on ND for a long period of time (exceeding 12 weeks) may arguably be more severe than the NAD^+^ deficiency measured in the 31 month-old mice. On the other hand, while the spermatogenic defects observed in these old mice may be less severe, they were clearly detectable and may at least in part be caused by NAD^+^ deficiency. Furthermore, the dynamics of human testicular NAD^+^ decline with age may be different from mice, along with its importance for human male fertility, which will require further research. Additional investigations are currently underway to determine the role of NAD^+^-decline in the aging process in ANDY mice. In summary, this study is the first one to show that experimentally induced low testicular NAD^+^ levels result in reversible disruption of spermatogenesis, adding vitamin B3 to the list of vitamins that are essential for proper spermatogenesis in humans. The study also provides clues to the role of NAD^+^ decline in the age-related decline of testicular function and male fertility.

## Data Availability Statement

The raw data supporting the conclusions of this article will be made available by the authors, without undue reservation.

## Ethics Statement

The animal study was reviewed and approved by Institutional Animal Care and Use Committees (IACUC) of Utah State University and of Mayo Clinic, Rochester, Minnesota.

## Author Contributions

MM-F and RM contributed conception and design of the study, experimentation, data analysis and preparation of the manuscript. AZ, CS, AT, SL, MW and HC performed experiments, collected and analysed data. GW, KT, CC, and EC contributed materials. All authors contributed to manuscript revision, read, and approved the submitted version.

## Funding 

Research reported in this publication was supported by the Eunice Kennedy Shriver National Institute Of Child Health & Human Development of the National Institutes of Health under Award Number HD100970 (to RM) and HD103027 (to MM-F) and by the National Institute On Aging of the National Institutes of Health under Award Number AG069745 (to MM-F and EC), and by a Utah Agricultural Experiment Station grant, Utah State University (UTA01403, to RM).

CS was supported by a Veterinary Student Summer Research Fellowship of the School of Veterinary Medicine, Utah State University.

## Author Disclaimer

The content is solely the responsibility of the authors and does not necessarily represent the official views of the National Institutes of Health.

## Conflict of Interest

The authors declare that the research was conducted in the absence of any commercial or financial relationships that could be construed as a potential conflict of interest.

## Publisher’s Note

All claims expressed in this article are solely those of the authors and do not necessarily represent those of their affiliated organizations, or those of the publisher, the editors and the reviewers. Any product that may be evaluated in this article, or claim that may be made by its manufacturer, is not guaranteed or endorsed by the publisher.

## Supplementary Material

Supplementary Figure 1 Time course of blood NAD levels in ANDY mice kept on indicated diets. Adult, sexually mature ANDY males ranging in age from 7-14 weeks were placed on indicated diets. Blood samples were taken prior to initiation of diet (pre-diet), after 2, 4, 6 and 11 weeks on diet. NAD content was determined using enzymatic cycling assays. Blood NAD levels decline between 2-6 weeks on diet. No further drop in blood NAD levels were observed between 6 and 11 weeks on diet. Significance of differences in A. was determined using 2-way ANOVA with Tukey’s multiple comparison, in B. 1-way ANOVA. **p < 0.01, ***p < 0.001, ****p < 0.0001.Letters a, b, c in figure B indicate significant changes with p-values in the *** to **** range. Identical letters indicate that there was no statistically significant difference.
