# Supplementary figures and images for: Low NAD+ Levels Are Associated With a Decline of Spermatogenesis in Transgenic ANDY and Aging Mice
Source: Front Endocrinol (Lausanne). 2022 May 6;13:896356. doi: 10.3389/fendo.2022.896356 (PMC9120959; doi:10.3389/fendo.2022.896356)

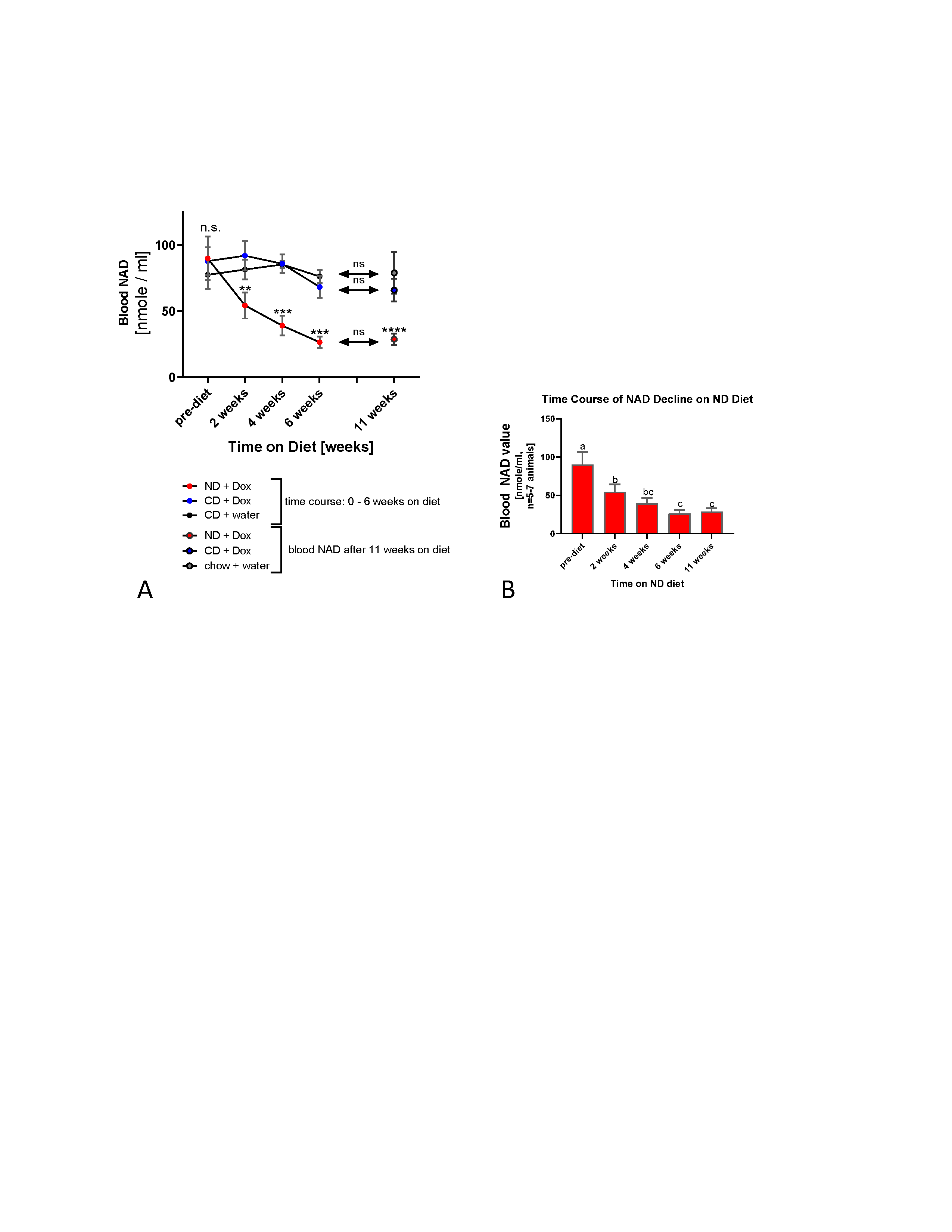

Supplement: Supplementary Figure 1 — Time course of blood NAD levels in ANDY mice kept on indicated diets. Adult, sexually mature ANDY males ranging in age from 7-14 weeks were placed on indicated diets. Blood samples were taken prior to initiation of diet (pre-diet), after 2, 4, 6 and 11 weeks on diet. NAD content was determined using enzymatic cycling assays. Blood NAD levels decline between 2-6 weeks on diet. No further drop in blood NAD levels were observed between 6 and 11 weeks on diet. Significance of differences in A. was determined using 2-way ANOVA with Tukey’s multiple comparison, in B. 1-way ANOVA. **p < 0.01, ***p < 0.001, ****p < 0.0001.Letters a, b, c in figure B indicate significant changes with p-values in the *** to **** range. Identical letters indicate that there was no statistically significant difference. [file Image_1.tif]
