# Supplementary material for: Low NAD+ Levels Are Associated With a Decline of Spermatogenesis in Transgenic ANDY and Aging Mice
Source: Front Endocrinol (Lausanne). 2022 May 6;13:896356. doi: 10.3389/fendo.2022.896356 (PMC9120959; doi:10.3389/fendo.2022.896356)
Supplement: Supplementary file 2 [file Table_1.docx]

**Supplemental Table 1 (to Figure 1A)**: **Coefficients of variation in quantification of testicular NAD values by enzymatic cycling assays.**
NAD was determined in testis tissue collected from seven individual mice per indicated feed group. Each testis sample lysate was measured in quadruplicate; measurements were distributed across three 96-well assay plates. Intra-assay coefficient of variation (CV) was determined within each feed group in each plate and the average CV across the plates was calculated. Inter-assay CV was calculated for each of the samples across the three plates. CV% was calculated by dividing the standard deviation by the mean, then multiplying by 100 to convert to a percentage. The overall CVs were determined across all samples for the intra assay CVs and inter assay CVs.

| Parameter | Diet groups | | Overall |
| --- | --- | --- | --- |
| Intra assay CV [%] | ND + Dox | 13.5% | 7.9% |
|  | CD + Dox | 4.6% |  |
|  | Chow + Dox | 5.9% |  |
|  | Chow + water | 6.6% |  |
| Inter assay CV [%] | ND + Dox | 10.5% | 9.0% |
|  | CD + Dox | 8.2% |  |
|  | Chow + Dox | 10.3% |  |
|  | Chow + water | 6.9% |  |
